# Financial impact of adopting implantable loop recorder diagnostic for unexplained syncope compared with conventional diagnostic pathway in Portugal

**DOI:** 10.1186/1471-2261-14-63

**Published:** 2014-05-06

**Authors:** Rui Providência, Rui Candeias, Carlos Morais, Hipólito Reis, Luís Elvas, Vitor Sanfins, Sara Farinha, Simon Eggington, Stelios Tsintzos

**Affiliations:** 1 Centro Hospitalar e Universitário de Coimbra, Serviço de Cardiologia, Coimbra, Portugal; 2 Faculdade de Medicina, Universidade de Coimbra, Coimbra, Portugal; 3 Hospital de Faro EPE, Faro, Portugal; 4 Hospital Prof. Doutor Fernando da Fonseca EPE, Amadora, Portugal; 5 Hospital de Santo António, Centro Hospitalar do Porto, Porto, Portugal; 6 Hospital de Guimarães, Centro Hospitalar do Alto Ave EPE, Guimarães, Portugal; 7 Medtronic, Lisboa, Portugal; 8 Medtronic, Tolochenaz, Switzerland; 9 Serviço de Cardiologia, Hospital Geral do, Centro Hospital e Universitário de Coimbra, Quinta dos Vales, 3041-801 S. Martinho do Bispo, Coimbra, Portugal

**Keywords:** Syncope, Implantable loop recorder, Emergency department, Budget impact analysis

## Abstract

**Background:**

To estimate the short- and long-term financial impact of early referral for implantable loop recorder diagnostic (ILR) versus conventional diagnostic pathway (CDP) in the management of unexplained syncope (US) in the Portuguese National Health Service (PNHS).

**Methods:**

A Markov model was developed to estimate the expected number of hospital admissions due to US and its respective financial impact in patients implanted with ILR versus CDP. The average cost of a syncope episode admission was estimated based on Portuguese cost data and landmark papers. The financial impact of ILR adoption was estimated for a total of 197 patients with US, based on the number of syncope admissions per year in the PNHS. Sensitivity analysis was performed to take into account the effect of uncertainty in the input parameters (hazard ratio of death; number of syncope events per year; probabilities and unit costs of each diagnostic test; probability of trauma and yield of diagnosis) over three-year and lifetime horizons.

**Results:**

The average cost of a syncope event was estimated to be between 1,760€ and 2,800€. Over a lifetime horizon, the total discounted costs of hospital admissions and syncope diagnosis for the entire cohort were 23% lower amongst patients in the ILR group compared with the CDP group (1,204,621€ for ILR, versus 1,571,332€ for CDP).

**Conclusion:**

The utilization of ILR leads to an earlier diagnosis and lower number of syncope hospital admissions and investigations, thus allowing significant cost offsets in the Portuguese setting. The result is robust to changes in the input parameter values, and cost savings become more pronounced over time.

## Background

Syncope represents a major health challenge for the medical team diagnosing its etiology, which can range from benign neurocardiogenic syncope to potentially fatal arrhythmias. The frequency of syncope episodes at the emergency department is reported in recent studies to range from 0.9% to 1.7% of all attendances [1-8]. Due to the potentially life-threatening etiology of syncope, patients often undergo a long and costly clinical pathway, which sometimes comprises extensive and repeated investigations. For this reason, syncope represents a clinical challenge and a financial burden to health care systems [9-11].

Evidence regarding the clinical and economic benefit of the implantable loop recorder (ILR) for the etiologic study of recurrent syncope has grown in recent years [12,13] and the use of such devices is currently supported by the 2009 European Society of Cardiology Syncope Guidelines [14].

Previous clinical studies using ILRs in Unexplained Syncope patients have focused on a 12-month period of time as opposed to a cohort-level analysis comparing ILR with non-ILR strategies over the lifetime of the patients [4,15,16]. Moreover, none of these analyses has been performed within the Portuguese healthcare setting.

We aimed to estimate the financial impact of adopting Implantable Loop Recorder Diagnostic for the diagnosis of unexplained syncope versus a conventional diagnostic pathway (CDP) in the context of the Portuguese National Health System.

## Methods

### Model

**Figure 1:**
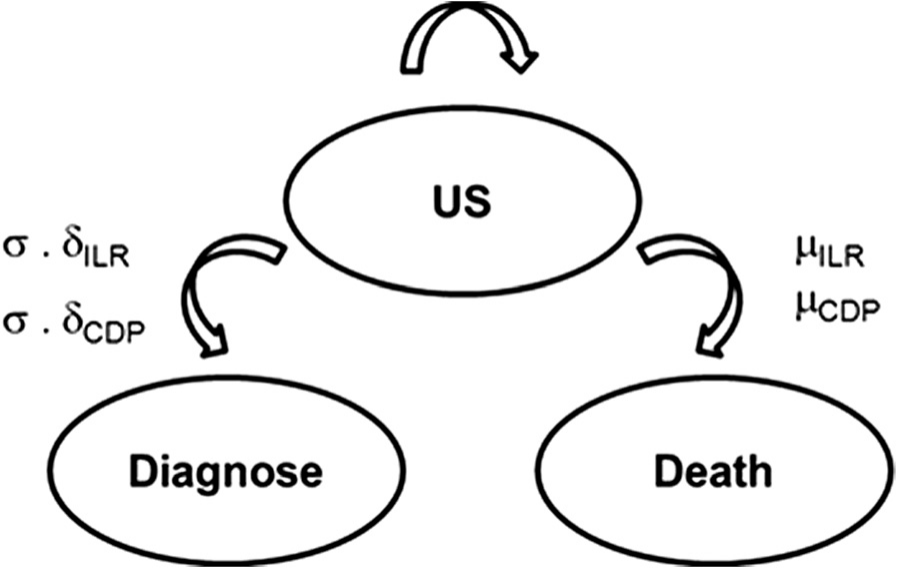
**Schematics of the model proposed.** The model considers two groups, Implantable Loop Recorder (ILR) and Conventional Diagnostic Pathway (CDP) groups, with different state-transition probabilities (μ,; σ, syncope; and δ, diagnosis). (US = undiagnosed syncope).

A discrete-time Markov chain was developed to estimate the expected number of hospital admissions due to unexplained syncope, and respective financial impact in patients implanted with ILR versus patients following the conventional diagnostic pathway, over 3-year and lifetime (30 years) time horizons. The 3-year period was chosen to reflect the current battery life of leading ILRs [17]. The model considered three health states (undiagnosed syncope, diagnosis and death), and two groups with different state transition probabilities – ILR and CDP (Figure 1). Each year, patients with undiagnosed syncope faced the probability of death, of a recurrent syncope event and in case of a syncope event, the probability of being diagnosed. Once a diagnosis was achieved (assuming that the patient is successfully treated) or death occurred, the patient exited the model and no further costs or syncope events were modelled. The state transition probabilities were taken from landmark papers as described below.

The probability of death in both patient groups was based on the Portuguese Population age–specific mortality rate [18]. Following Soteriades et al. [11], this rate was adjusted by a factor of 1.32 to take into consideration the adjusted hazard ratio for the risk of death from any cause in patients with unexplained syncope. We applied a rate of 0.6 syncopes per year among undiagnosed patients in both arms (the value was adjusted to the 3-month cycle length used), based on follow-up data from Farwell [19]. This is consistent with data from Brignole, who reported a mean of 0.83 syncopes per year during follow-up [20]. This is a conservative approach, since a higher syncope recurrence rate would favour ILR due to its improved diagnostic power.

The probability of diagnosis should a recurrent syncope event occur was modelled on a probability-per-event basis – we used 62.8% for the ILR arm, based on data on the rate of successful ECG capture (either automatic or manual from the device after a syncope event) in the EaSyAS study reported by Farwell [19] (27 patients out of 43 were successfully diagnosed). This diagnosis rate was applied only in the first three years of the model, to reflect the battery lifetime of the ILR. In the CDP group, a diagnosis rate per syncope of 12.5% was used, also based on data from Farwell [19] (4 out of 32 patients were diagnosed in the control arm). This diagnosis rate was also applied beyond 3 years in the ILR group, due to exhaustion of the ILR battery – the assumption therefore is that the ILR will not be replaced if a diagnosis has not been achieved within three years of implantation. We used the EaSyAS study as the source for these parameters to avoid introducing bias through use of data from different studies, even though a more recent study included the latest version of the ILR, which has significant algorithm improvements and better performance (e.g. older-generation devices sometimes failed to auto-activate) [21]. Sensitivity analyses were undertaken to explore the impact of different data sources for the incidence of recurrent syncope and the probability of diagnosis.

The model then dynamically calculated the number of patients diagnosed in each cycle, multiplying the number of syncopes by the probability of obtaining a diagnosis should syncope recur. Generally speaking, this approach can increase the validity of the sensitivity analyses since both critical variables (rate of syncope recurrence and probability of successful ECG capturing) can be tested and varied independently of each other. A cycle length of 3 months was used in the model, thus ensuring the possibility that a patient suffers more than one event per year.

### Study population

Our study population was composed of patients with unexplained and recurrent syncope leading to a Hospital or Emergency Department admission, with an average starting age of 61 years old, following the patient population characteristics from the recent PICTURE study, the largest international study using ILR regarding the type and frequency of investigations that are usually performed in the standard diagnostic pathway [21].

Unexplained syncope was defined as a syncopal event whose etiology was not clarified after a clinical history, physical examination and 12-lead ECG. In order to define the number of patients to consider in the model we used the latest National official Diagnosis-Related Groups (DRG) Report published by the Central Administration of Health System (ACSS) and assumed the number of syncope events remained constant in recent years. According to this report there were a total of 1010 hospital admissions, either in an inpatient or ambulatory setting due to syncope (DRG 141 and 142) in the Portuguese National Health System Hospitals in 2006 [22]. From 1010 hospital admissions due to syncope, we assumed that 19.5% were due to unexplained and recurrent syncope [21,23]. Consequently the analysis considered a sample of 197 patients for each of the treatment groups being compared.

Sources used were published investigations (approved by the authors’ institutional Ethics Committees) and data from National regulatory authorities. No human participants were directly included or recruited in this study. Only a model/simulation was built according to the available data/evidence for this population.

### Costs

In order to provide an estimation of the average cost of a syncope episode admission, we have considered five scenarios, using data on the frequency of a range of diagnostic rests from the various studies recently published on syncope management (Sousa Pedro [24] Edvardsson [21]; Baron-Esquiviais [7]; Brignole [4]; Farwell [16]) to which we applied the unit costs/prices published by the Portuguese National Health Service prices table [25]. Using a conservative approach, we decided to exclude from the analysis the exams for which Portuguese unitary costs were not available. The average diagnostic cost per syncope was determined to be between 72.41€ and 1,112.02€ (see details in Table 1); in the base-case analysis, a diagnostic cost of 164.32€ was applied (based on Sousa Pedro [24]) to all syncope events in the CDP arm (regardless of whether a successful diagnosis was made), and to syncope events occurring in the ILR arm beyond the 3-year device battery life.

**Table 1:** Average cost of diagnostic exams per patient with syncope

**Investigations **[21]	**Unit prices**	**Sousa Pedro **[24]		**Edvardsson **[21]		**Baron-Esquivias **[7]		**Brignole **[4]		**Farwell **[16]		**Unit prices reference **[25]
		**Percentage of patients**	**Weighted value**	**Percentage of patients**	**Weighted value**	**Percentage of patients**	**Weighted value**	**Percentage of patients**	**Weighted value**	**Percentage of patients**	**Weighted value**	
Standard electrocardiogram	6.50€	100%	6.50€	98%	6.37€	95,6%	6.21€	100%	6.50€	-	-	Code 40301
Echocardiography	53.20€	72.2%	38.41€	86%	45.75€	2,1%	1.12€	16%	8.51€	15.31%	8.14€	Code 40550
Overload echocardiography	85.30€	1.60%	1.36€		-	-	-		-		-	Code 40550;40315
Abdominal echography	20.12€	-	-	-	-	-	-	2%	0.40€	-	-	Code 17130
Basic laboratory tests		-	-							-	-	Pack Estimation^a^
With/enzymes tests	65.87€			86%	56.65€			35%	23.05€			
Without enzymes tests	53.87€					70,2%	37.82€					
Enzymes^b^	34.40€	-	-	-	-	30,2%	10.39€	-	-	-	-	Pack Estimation^b^
Ambulatory ECG monitoring	43.70€	-	-	67%	29.28€	-	-			11.22%	4.91€	Code 40405
ELR	47.30€	-	-	-	-	-	-	-	-	28.57%	13.51€	
In-Hospital ECG monitoring	124.70€	61.20%	76.32€	55%	68.59€	17,1%	21.32€	11%	13.72€	-	-	Code 40495 + daily admission (85€)
Exercise testing	32.10€	19.60%	6.29€	52%	16.69€	-	-	3%	0.96€	-	-	Code 40315
MRI or CT scan	97.45€	1.60%	1.56€	47%	45.80€	-	-	15%	14.62€	-	-	Codes 18010; 16010
MRI	127.90€	-	-	-	-	-	-	-	-	1.02%	1.31€	Code 18010
Brain CT scan	67.00€	-	-	-	-	9%	6.03€	-	-	8.16%	5.47€	Code 16010
Thorax CT scan	74.70€	-	-	-	-	1,1%	0.82€	-	-	-	-	Code 16060
Chest X-ray	9.00€	-	-	-	-	51,9%	4.67€	12%	1.08€	-	-	Code 10406
Electroencephalography	58.80€	1.20%	0.71€	39%	22.93€	-	-	6%	3.53€	2.04%	1.20€	Code 63010
Carotid sinus massage	6.50€	2.90%	0.19€	36%	2.34€	0,5%	0.03€	15%	0.98€	-	-	Code 40301
Carotid echo-doppler	23.17€	12.70%	2.94€	-	-	-	-	4%	0.93€	5.10%	1.18€	Code 17290
TILT test	124.10€	15.90%	19.73€	35%	43.44€	-	-	13%	16.13€	-	-	Code 41120
Electrophysiology testing	2,488.72€	-	-	25%	622.18€	-	-	3%	74.66€	1.02%	25.40€	Code 40950
Coronary angiography	531.44€	-	-	23%	122.23€	-	-	2%	10.63€	-	-	Code 40820
External loop recording	47.30€	11.80%	5.58€	12%	5.68€	-	-	-	-	-	-	Code 40479
Orthostatic blood pressure movements	4.00€	-	-	48%	1.92€	4,6%	0.18€	-	-	-	-	Code 99230
Hypertension map	59.20€	6.90%	4.08€	-	-	-	-	-	-	-	-	Code 41010
Neurological or psychiatric evaluation	30.90€	-	-	47%	14.52€	-	-	-	-	-	-	Code 82040
Adenosine Triphosphate (ATP) test	Not available	-	-	3%	-	-	-	-	-	-	-	Not considered
Others	-	-	-	9%	-	7,20%	-	13%	-	-	-	Not considered
Total cost	-	-	164.32€	-	1,112.02€		88.51€	**-**	173.31€	**-**	72.41€	

^a^Basic Laboratory costs were estimated considering a standard pack of analysis: Creatinine, Hemogram with leukocyte formula, urea, capillary blood glucose determination, Ionogram (Na, K, Cl), Proteins (total), Albumin Ultra-sensitive C-reactive protein, Summary analysis of Urine, acid-base balance (pH, pCO2, pO2, SatO2, CO2), D- Dimer (DD); and prothrombin time (TP, Quick, INR), (with or without: Troponin T or I, alanine aminotransferase (ALT), aspartate aminotransferase (AST)).

^b^Enzymes tests: Troponin T or I, Creatine kinase (CK), Myoglobin (Mb), alanine aminotransferase (ALT), aspartate aminotransferase (AST) and lactic dehydrogenase (LDH).

Costs were taken from the Portuguese National Health Service Prices Table [25].

In addition to this cost, we estimated the cost of trauma/injury in patients suffering recurrent syncope. Due to a lack of Portuguese micro-costing data regarding trauma and injury in syncope patients, we used two DRG tariffs as a proxy for the cost of injury/trauma in an inpatient setting. These DRGs were considered since, although not referring to episodes where syncope is the main diagnosis, they refer to episodes where syncope is coded as a secondary diagnosis and reflect the resource consumption regarding injury/trauma in these patients. According to Sousa Pedro et al. [24], 52.2% of syncope episodes are associated with trauma, while Bartolleti et al. [26] reports that 16.16% of traumas/injuries are severe. Combining these figures with the selected DRG tariffs for minor and major trauma (2,684.83€ and 6,058.25€, respectively – see Table 2), gives an average cost of injury/trauma of 1,687.57€ per syncope event.

**Table 2:** **Injury/trauma diagnosis-related groups **[25]

**DRG**	**Description**	**Tariff **[25]	**Occurrence of minor/major trauma **[24] [26]	**Injury/trauma due to syncope average cost estimation**
767	Stupor and/or traumatic coma, coma <1 hour age > 17 years without CC	2,684.83 €	52.2% *(1-16.16%) = 43.8%	1,687.57 €
468	Extensive procedures in the Operating Room, unrelated to principal diagnosis	6,058.25 €	52.2% *16.16% = 8.45%	

Legend: DRG – Diagnosis-Related-Group; CC- Complications/comorbidities.

“*” means “multiply”. E.g. 52.2% multiplied by (100-16.16%).

The costs associated with the ILR were accounted for in four ways: device acquisition, implantation, follow-up and explantation. The acquisition cost of the ILR was considered to be 2,000€ (based on the price of the Reveal® DX loop recorder in Portugal), which is in line with the studies reported by Davis et al., [27,28] to which a device implantation cost of 127.80€ was added, based on the Portuguese National Health Service official prices table (Code 41395) [25]. Further, it was assumed that patients with an active device would require two device check visits per year, using the official tariff for a medical consultation of 31€ per visit [25]. Finally, the cost of device explantation was applied at the time of diagnosis or after three years – this cost was set equal to the cost of implantation. This last cost was not applied to patients who died within the first three years of the model.

All costs were discounted at 5% per year, in accordance with the Portuguese Guidelines for Economic Drug Evaluation Studies [29].

### Scenarios presented

The primary outputs from this analysis were the number of diagnosed patients and the cost of syncope admissions and diagnosis in both groups (ILR and CDP). The model was run over both lifetime and 3-year time horizons to illustrate the cost profile over time. One-way sensitivity analysis was undertaken to explore the impact of uncertainty in specific model parameters and to use data from alternative sources. Probabilistic sensitivity analysis was also performed, incorporating uncertainty in the following parameters: hazard ratio of death; number of syncope events per year; probability and unit cost of each diagnostic test; cost of device acquisition, implantation, follow-up and explantation; probability and cost of major and minor trauma; diagnostic yield of each diagnostic pathway. In the probabilistic analysis, uncertainty was characterised by specifying a probability distribution for each model parameter. The probability distributions used were selected to be appropriate for the type of parameter e.g. lognormal distribution for the hazard ratio of death and the syncope recurrence rate, gamma distributions for all cost parameters and beta distributions for all probability-based parameters (probabilities of syncope diagnosis and of the use of each diagnostic test). Monte-Carlo simulation was then used to sample a value from each parameter’s distribution and propagate the uncertainty through the model to generate a set of plausible outputs. Ten thousand simulations were undertaken to ensure that the effect of the uncertainty was fully captured. The probabilistic analysis was repeated for each of the five scenarios, using different study data for the incidence of different diagnostic tests in the CDP arm. A summary table of the parameters of each probability distribution is provided in the Additional file 1.

## Results

### Deterministic analysis

After running the model for both three-year and lifetime horizons it was possible to estimate the financial impact for the two patient groups (see Table 3). From 197 Patients in the ILR group, 135 (68.5%) were diagnosed within a 3-year period and of these, 64 (47.4%) were diagnosed in the first year. By contrast, 40 (20.3%) of the patients from the CDP group were diagnosed in the first three years. The total cost of hospital admissions in a three-year time horizon due to syncope was 23% lower in the ILR group than in the CDP Group in the base-case analysis, after factoring in the costs of diagnostic tests in the CDP group (1,204,621€ for the ILR group, versus 1,571,332€ for the CDP group - this translates to a saving of 1,861€ per patient). In all four of the other main scenarios tested (using diagnostic test frequency data from other studies), ILR led to an earlier diagnosis and consequently to a lower number of syncope hospital admissions, allowing important hospital cost offsets, with savings of between 307,872€ and 973,429€ for the entire cohort (1,563€–4,941€ per patient) over a lifetime horizon (Table 3). Over a 3-year horizon, costs were higher in the ILR group, due to the up-front cost of device acquisition and implantation.

**Table 3:** Number of syncope hospital admissions, diagnosed patients and costs for ILR and CDP groups in different scenarios

**Model output**	**ILR**		**CDP**		**Overall savings**	
**Time horizon**	**Lifetime**	**3 years**	**Lifetime**	**3 years**	**Lifetime**	**3 years**
Number of patients diagnosed	175	135	143	40	32	95
Number of patients undiagnosed and alive	1	58	2	152	-1	-94
Total syncope episodes	531	215	1,141	315	610	100
Number of injuries (major and minor)	278	112	596	165	318	53
Costs – device acquisition	394,000€	394,000€	0€	0€	-394,000€	-394,000€
Costs – device-related expenses	71,101€	71,101€	0€	0€	-71,101€	-71,101€
Costs – syncope admissions	705,292€	353,756€	1,431,910€	513,879€	726,618€	160,123€
Costs – diagnostic tests*	34,228€	0€	139,422€	50,035€	105,194€	50,035€
Costs – total (Sousa Pedro)	1,204,621€	818,857€	1,571,332€	563,915€	366,711€	-254,943€
Costs – total (Edvardsson)	1,402,037€	818,857€	2,375,465€	852,499€	973,429€	33,642€
Costs – total (Baron-Esquivias)	1,188,830€	818,857€	1,507,011€	540,831€	318,181€	-278,026€
Costs – total (Brignole)	1,206,495€	818,857€	1,578,965€	566,654€	372,470€	-252,203€
Costs – total (Farwell)	1,185,476€	818,857€	1,493,347€	535,928€	307,872€	-282,929€

*Diagnostic test costs calculated from resource use data reported by Sousa Pedro [24]. The ‘total’ results for Edvardsson, Baron-Esquivias, Brignole and Farwell instead use the diagnostic test resource use data from the respective studies.

### One-way sensitivity analysis

**Figure 2:**
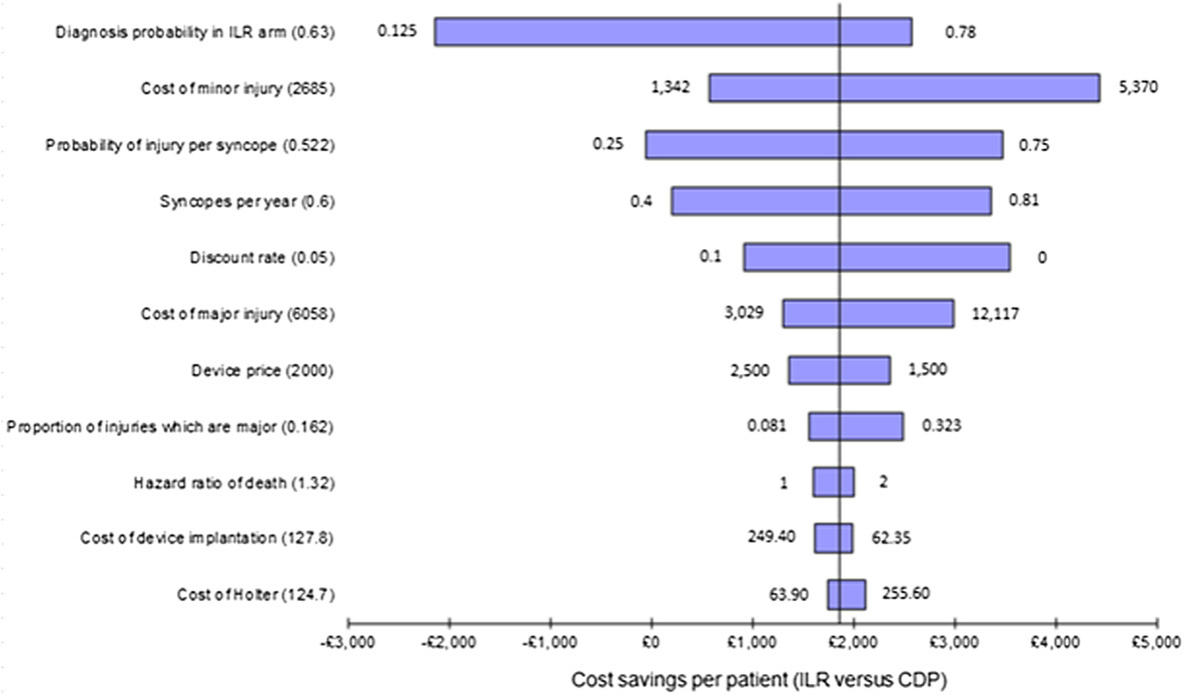
**Tornado diagram (one-way sensitivity analysis).** Numbers in brackets represent the deterministic value of each parameter; the numbers at each end of the bars represent the lower and upper bounds of the value used for each parameter.

**Figure 3:**
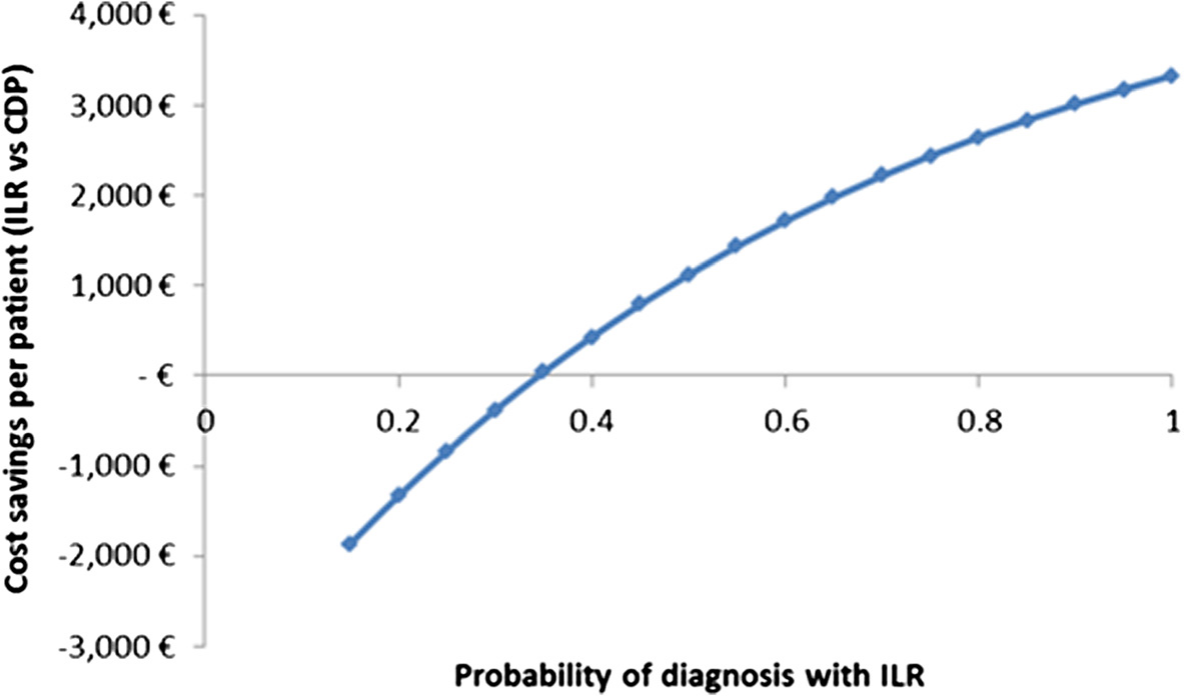
One-way sensitivity analysis – relationship between ILR diagnosis probability and lifetime cost savings.

The results of the one-way sensitivity analysis are shown in Figure 2. The x-axis represents the per-patient cost savings in the ILR arm over a lifetime horizon. The vertical line in the centre of the chart represents the deterministic cost savings for the ILR group, the bars towards the top of the chart show the parameters whose uncertainty has the most influence upon this outcome (the numbers at either end of each bar represent the range of values used for each input parameter), and the values in brackets show the base-case value of each input parameter. The ranges used for each parameter were specified either by using data from other studies or by halving (or doubling) the base-case values (for example, the upper bound of 0.78 used for the diagnosis probability in the ILR arm was based on PICTURE; the upper bound of 0.81 for the number of syncopes per year was reported by an earlier publication of the EaSyAS study (Farwell, (16)). Based on this analysis, the key parameters appear to be: the diagnosis probability in the ILR arm; the cost per syncope-related minor injury; the probability of injury per syncope event; and the syncope rate per year. ILR is projected to provide cost savings in all but two scenarios; the first of these involved reducing the probability of injury to 0.25 (from 0.522 in the base-case), while the second involved setting the probability of diagnosis in the ILR arm to be equal to that in the CDP arm, representing an extremely conservative approach. To explore this latter scenario more thoroughly, we examined the relationship between the probability of diagnosis with ILR and the overall cost savings predicted by the model. This relationship is shown in Figure 3 and indicates that with our model, provided that the probability of diagnosis with ILR is above 35% and the absolute incremental probability of diagnosis versus CDP is at least 17.5%, ILR is projected to provide cost savings compared with CDP. None of the diagnostic test costs had a significant impact upon the cost savings calculated from the model.

### Probabilistic sensitivity analysis

**Figure 4:**
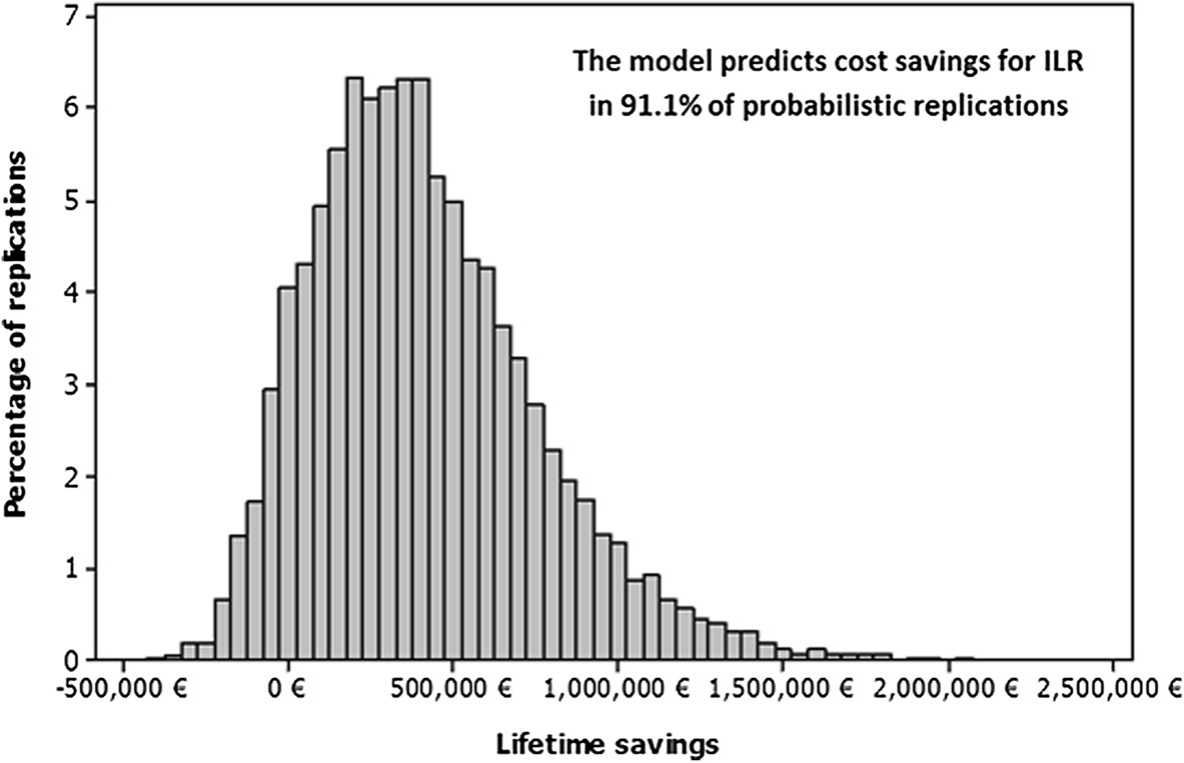
Probabilistic analysis results - lifetime savings for ILR.

The probabilistic analysis was performed initially using the resource use assumptions from Sousa et al. [24]. Based on the set of 10,000 sampled input parameter values, the mean lifetime savings for the ILR group compared with the CDP group were 411,167€ for the entire cohort (or 2,087€ per patient). Figure 4 shows a histogram of the cost savings (ILR versus CDP) for the 10,000 probabilistic runs – it can be observed that the significant majority (91.1%) of these resulted in cost savings in the ILR group. Probabilistic analyses were also performed under the four other scenarios (using resource data from different studies). In each case, at least 87% of simulations resulted in cost savings in the ILR group over a lifetime horizon.

## Discussion

We used a Markov Model based on government data and landmark international papers to compare the cost of ILR use versus CDP in unexplained recurrent syncope patients. Our results demonstrate that the use of ILR leads to fewer hospital admissions and investigations, suggesting its potential for significant cost offsets in the Portuguese National Health Service.

We have considered five scenarios based on data from different studies recently published in this subject area. We consider PICTURE [21] to be the most appropriate scenario since the study population enrolled most closely resembles ILR-indicated patients and thus the diagnostic tests observed are the ones with a higher likelihood of resembling the tests performed on unexplained syncope patients. The remaining scenarios used data from studies that focused generally on syncope patients and therefore included patients not indicated for ILR. Nevertheless, we believe there is value in testing all possible scenarios. Although PICTURE seems to better reflect the study population, we have used Sousa Pedro study as our base-case scenario, since it reports data from a Portuguese Population.

Under every scenario considered, ILR appears to be significantly cost-saving over a lifetime horizon. One-way sensitivity analyses failed to identify any plausible scenario under which ILR use does not result in significant cost-savings, prompting us to believe that ILRs should be offered to indicated patients. Probabilistic sensitivity analysis suggested that ILR is very likely to be cost-saving over a lifetime horizon, based on the modelling approach used and specification of parameter uncertainty.

The diagnosis rates predicted by the model closely match the data reported by Farwell et al. [16] and are aligned with the observations of Krahn et al. [12] Longer-term projections of the proportion of patients diagnosed also support long-term follow-up of the ISSUE-2 study, in which it has been projected that 80% of patients with an ILR would be diagnosed within four years [30].

The discrepancy between the guideline indications for the implantation of ILRs and their use in clinical practice has been described by Vitale E et al. [31] for the Italian population. Nevertheless, we highlight than in the 2005 to 2007 period, ILR implants in Italy were approximately three times more frequent (10 to 20 per million inhabitants) than in Portugal, where a very low number of implants has been observed: six ILRs per million inhabitants were implanted in 2011 [32]. This was about four to five times less than in Western Europe (15 to 28 ILRs per million inhabitants) and eight to ten times less than in the United Kingdom (28 to 59 ILRs per million inhabitants) [33], and well below the EHRA recommended rate of 135 implants/million [17].

If a total of 197 patients with unexplained syncope, as considered in this analysis, were implanted with ILR per year this would lead to an implant rate of 20 ILRs per million inhabitants, which would be in line with the practice from other European countries and would lead to considerable cost offsets to the Portuguese National Health Service. According to these results, limiting the use of ILRs in patients with a clear clinical indication leads to an increase in the number of other investigations and avoidable injuries. This in turn results in an increase of expenditure compared with other European countries. Furthermore, in the Portuguese context the use of ILR seems to occur in a late stage of the diagnostic workup of syncope. This suggests that patients have probably already undergone an exhaustive assessment with many investigations; this could mean that these patients have already used a lot of valuable healthcare resources which could have been invested elsewhere if ILR had been used earlier in the diagnostic pathway.

Previous studies on the use of ILR in a short term period have also demonstrated a favourable cost-benefit ratio. In the Randomized Assessment of Syncope Trial (RAST) ILR usage was compared with a conventional strategy (including the use of external loop recorder during 2 to 4 weeks, TILT testing and electrophysiological study - EPS) and achieved a higher frequency of diagnosis in subjects with unexplained syncope (55% versus 19%; p = 0.0014) [12]. The same study demonstrated the superior cost-effectiveness performance of the ILR strategy translated by a reduced cost-per-successful-diagnosis using ILRs; 5,852$ CAD for each diagnosis versus 8,414$ CAD in the conventional strategy group [15].

In the Eastbourne Syncope Assessment Study (EaSyAS) a further evaluation of the impact of the ILR compared to conventional investigation was performed. The invasive strategy achieved earlier, more diverse and more frequent (hazard ratio 8.93 95% CI 3.17–25.2, p < 0.0001) diagnosis, resulting in cost savings (mean difference of £809) [19]. Additionally, the diagnostic accuracy, safety, reliability and usefulness of the ILR has already been demonstrated in recent trials and in specific populations, such as patients with congenital heart disease [21,34,35] and patients undergoing magnetic resonance imaging [36].

### Study limitations

Incidence of DRG codes to estimate the size of the population may be associated with some error. Underestimation may occur in patients admitted with trauma due to syncope, if the type of lesion is codified as the main diagnosis and syncope is not introduced/codified. Conversely, if patients were admitted twice in the same year, that may have led to some degree of overestimation of the total number of patients. However, we think that these events may correspond to a minority of cases and may have partially and mutually neutralized themselves, since one led to overestimation and the other to underestimation.

We do not know for sure the Portuguese reality regarding the number and type of investigations that are performed before obtaining a diagnosis of referral of the patient to an ILR, namely the number of 24-hour Holter monitoring tests, external loop recorders, transthoracic echocardiogram, blood tests, head Computed Tomography scans and coronary angiograms exams. Sousa and colleagues report data from a syncope unit in the south of Portugal, and although it refers specifically to Portuguese practice in syncope management, it is a very unique case in the country and therefore may not reflect the national reality [24]. Nevertheless, taking into account the very low incidence of ILR implants in our country, a possible deviation is for having a higher number and more diverse type of investigations in Portugal, which would actually underestimate the expenditure with investigations, leading to a more favourable cost profile for ILR.

For the diagnostic yield, we used assumptions based on the EaSyAS study [19] for both treatment groups. EaSyAS was conducted with an earlier ILR device version, while the more recent PICTURE study was conducted with ILRs which have vastly improved auto-detection algorithms [13,21]. Since the devices currently on the market are DX/XT we consider the base-case results described here to be represent conservative estimates of the savings possible through wider use of ILRs. Furthermore, our study considered only the initial costs of injuries sustained as a result of syncope episodes (using the tariffs for minor and major injuries). This is likely to under-estimate the total costs of injury management, since it does not account for the ongoing costs of managing serious injuries and long-term sequelae [37]. Bartoletti reports that the majority of syncope-related traumas are to the head, suggesting that managing these events would in many cases involve greater costs than that of the initial hospitalisation [26].

## Conclusions

These results demonstrate that the use of an implantable loop recorder early in the assessment of patients with recurrent unexplained syncope has a favourable cost profile in the Portuguese context.

Based on the very low utilization of these devices in Portugal when compared with most European countries, we can assume that a stricter compliance with the 2009 ESC syncope guidelines may lead to an earlier use and important reduction in health care expenditure associated with unexplained recurrent syncope patients.

## Competing interests

RP has research grant from Medtronic; SF, ST and SE are employees of Medtronic; There are no conflicts of interest among the other authors.

## Authors’ contributions

RP, SF and ST have prepared the first draft of the article, which was revised by all authors. RP, RC, CM, HR, LE and VS, revised the used data in the models, conferring its adequateness for the Portuguese setting. All authors prepared insight and suggestions for the preparation of the final version of the manuscript. This version was revised once more by all the authors and approved before submission. Besides revising the manuscript, SF, ST and SE also provided statistical review for the used models.

## Pre-publication history

The pre-publication history for this paper can be accessed here:

http://www.biomedcentral.com/1471-2261/14/63/prepub

## Supplementary Material

Additional file 1 Brief summary of the parameters of each probability distribution. Click here for file
