# Supplementary material for: Financial impact of adopting implantable loop recorder diagnostic for unexplained syncope compared with conventional diagnostic pathway in Portugal
Source: BMC Cardiovasc Disord. 2014 May 6;14:63. doi: 10.1186/1471-2261-14-63 (PMC4101834; doi:10.1186/1471-2261-14-63)
Supplement: Additional file 1 — Brief summary of the parameters of each probability distribution. [file 1471-2261-14-63-S1.docx]

| **Parameter** | **Distribution type** | **Distribution type and parameters** | **Source / comment** |
| --- | --- | --- | --- |
| Hazard ratio of death for patients in model (versus age-matched population) | Lognormal | Log mean = 0.278; log SD = 0.0995. Distribution mean = 1.32 | Soteriades (2002) |
| Number of syncopes per year (ILR group) | Lognormal | Log mean = -0.511; log SD = 0.158. Distribution mean (exponentiated) = 0.6 | Farwell (2004). 95% confidence interval based on 30% variation either side of the mean |
| Probability of diagnosis for syncopes occurring during first 3 years (ILR group) | Beta | Alpha = 27; Beta = 16. Distribution mean = 0.628 | Farwell (2004) |
| Probability of diagnosis for syncopes occurring beyond 3 years or in the control group | Beta | Alpha = 4; Beta = 28. Distribution mean = 0.125 | Farwell (2004) |
| Probability of injury per syncope | Beta | Alpha = 128; Beta = 117. Distribution mean = 0.522 | Sousa (2013) |
| Cost per minor injury (trauma) following syncope | Gamma * | Alpha = 42.68; Beta = 62.9. Mean of distribution = 2,685 | 95% confidence interval constructed assuming 30% variation either side of the mean |
| Cost per major injury (trauma) following syncope | Gamma * | Alpha = 42.68; Beta = 141.93. Mean of distribution = 6,058 | 95% confidence interval constructed assuming 30% variation either side of the mean |
| Proportion of injuries which are major | Beta | Alpha = 59; Beta = 306. Mean of distribution = 0.162 | Bartoletti (2008) |
| Cost of ILR | Gamma * | Alpha = 42.68; Beta = 46.86. Mean of distribution = 2,000 | Reveal® DX loop recorder. Portuguese price |
| Cost of ILR implantation | Gamma * | Alpha = 42.68; Beta = 2.99. Mean of distribution = 127.80 | Diàrio da República. Code 41395 - event recorder implantation |
| Cost of ILR monitoring (per visit) | Gamma * | Alpha = 42.68; Beta = 0.726. Mean of distribution = 31 | Medical consultation coding Article 15º, nº1 a) Law 163/2013 |
|  |  |  |  |
| Cost of ECG | Gamma * | Alpha = 42.68; Beta = 0.152. Mean of distribution = 6.50 | Diàrio da República. Code 40301 |
| Cost of echocardiography | Gamma * | Alpha = 42.68; Beta = 0.152. Mean of distribution = 53.20 | Diàrio da República. Code 40550 |
| Cost of overload echocardiography | Gamma * | Alpha = 42.68; Beta = 0.152. Mean of distribution = 85.30 | Diàrio da República. Codes 40550;40315 |
| Cost of Holter monitor | Gamma * | Alpha = 42.68; Beta = 0.152. Mean of distribution = 124.70 | Diàrio da República. Code 40495, plus daily admission (85€) |
| Cost of exercise test | Gamma * | Alpha = 42.68; Beta = 0.152. Mean of distribution = 32.10 | Diàrio da República. Code 40315 |
| Cost of CT / MRI | Gamma * | Alpha = 42.68; Beta = 0.152. Mean of distribution = 97.45 | Diàrio da República. Codes 18010;16010 |
| Cost of electroencephalogram | Gamma * | Alpha = 42.68; Beta = 0.152. Mean of distribution = 58.8 | Diàrio da República. Code 63010 |
| Cost of carotid sinus massage | Gamma * | Alpha = 42.68; Beta = 0.152. Mean of distribution = 6.50 | Diàrio da República. Code 40301 |
| Cost of carotid echo-Doppler | Gamma * | Alpha = 42.68; Beta = 0.152. Mean of distribution = 23.17 | Diàrio da República. Code 17290 |
| Cost of tilt test | Gamma * | Alpha = 42.68; Beta = 0.152. Mean of distribution = 124.10 | Diàrio da República. Code 41120 |
| Cost of ambulatory Holter monitor | Gamma * | Alpha = 42.68; Beta = 0.152. Mean of distribution = 47.30 | Diàrio da República. Code 40405 |
| Cost of hypertension map | Gamma * | Alpha = 42.68; Beta = 0.152. Mean of distribution = 59.20 | Diàrio da República. Code 41010 |
| Cost of basic lab tests | Gamma * | Alpha = 42.68; Beta = 0.152. Mean of distribution = 65.87 | Pack estimation |
| Cost of ambulatory ECG monitoring | Gamma * | Alpha = 42.68; Beta = 0.152. Mean of distribution = 43.70 | Diàrio da República. Code 40405 |
| Cost of electrophysiology testing | Gamma * | Alpha = 42.68; Beta = 0.152. Mean of distribution = 2488.72 | Diàrio da República. Code 40950 |
| Cost of coronary angiography | Gamma * | Alpha = 42.68; Beta = 0.152. Mean of distribution = 531.44 | Diàrio da República. Code 40820 |
| Cost of orthostatic blood pressure movements test | Gamma * | Alpha = 42.68; Beta = 0.152. Mean of distribution = 4 | Diàrio da República. Code 99230 |
| Cost of neurological / psychiatric evaluation | Gamma * | Alpha = 42.68; Beta = 0.152. Mean of distribution = 30.90 | Diàrio da República. Code 82040 |
| Cost of basic blood chemistry | Gamma * | Alpha = 42.68; Beta = 0.152. Mean of distribution = 53.87 | Pack estimation |
| Cost of enzymes | Gamma * | Alpha = 42.68; Beta = 0.152. Mean of distribution = 34.40 | Diàrio da República. Code Pack estimation |
| Cost of brain CT scan | Gamma * | Alpha = 42.68; Beta = 0.152. Mean of distribution = 67 | Diàrio da República. Code 16010 |
| Cost of thorax CT scan | Gamma | Alpha = 42.68; Beta = 0.152. Mean of distribution = 74.70 | Diàrio da República. Code 16060 |
| Cost of chest x-ray | Gamma * | Alpha = 42.68; Beta = 0.152. Mean of distribution = 9 | Diàrio da República. Code 10406 |
| Cost of abdominal echography | Gamma * | Alpha = 42.68; Beta = 0.152. Mean of distribution = 20.12 | Diàrio da República. Code 17130 |
| Cost of MRI | Gamma * | Alpha = 42.68; Beta = 0.152. Mean of distribution = 127.90 | Diàrio da República. Code 18010 |
| Probability of ECG | Beta | Alpha = 245.5; Beta = 0.5. Mean of distribution = 0.998  Alpha = 556; Beta = 14. Mean of distribution = 0.975  Alpha = 1163; Beta = 54. Mean of distribution = 0.956  Alpha = 745.5; Beta = 0.5. Mean of distribution = 0.999 | Sousa (2013)  Edvardsson (2010)  Baron-Esquivas (2010)  Brignole (2006) |
| Probability of echocardiography | Beta | Alpha = 177; Beta = 68. Mean of distribution = 0.722  Alpha = 490; Beta = 80. Mean of distribution = 0.86  Alpha = 25; Beta = 1192. Mean of distribution = 0.021  Alpha = 120; Beta = 625. Mean of distribution = 0.161  Alpha = 15; Beta = 83. Mean of distribution = 0.153 | Sousa (2013)  Edvardsson (2010)  Baron-Esquivas (2010)  Brignole (2006)  Farwell (2004) |
| Probability of overload echocardiography | Beta | Alpha = 4.5; Beta = 241.5. Mean of distribution = 0.0183 | Sousa (2013) |
| Probability of Holter monitor | Beta | Alpha = 150; Beta = 95. Mean of distribution = 0.612  Alpha = 311; Beta = 259. Mean of distribution = 0.546  Alpha = 208; Beta = 1009. Mean of distribution = 0.171  Alpha = 84; Beta = 661. Mean of distribution = 0.113 | Sousa (2013)  Edvardsson (2010)  Baron-Esquivas (2010)  Brignole (2006) |
| Probability of exercise test | Beta | Alpha = 48; Beta = 197. Mean of distribution = 0.196  Alpha = 297; Beta = 273. Mean of distribution = 0.521  Alpha = 23; Beta = 722. Mean of distribution = 0.031 | Sousa (2013)  Edvardsson (2010)  Brignole (2006) |
| Probability of CT / MRI | Beta | Alpha = 4.5; Beta = 241.5. Mean of distribution = 0.0183  Alpha = 267; Beta = 303. Mean of distribution = 0.468  Alpha = 115; Beta = 630. Mean of distribution = 0.154 | Sousa (2013)  Edvardsson (2010)  Brignole (2006) |
| Probability of electroencephalogram | Beta | Alpha = 3.5; Beta = 242.5. Mean of distribution = 0.998  Alpha = 222; Beta = 348. Mean of distribution = 0.389  Alpha = 42; Beta = 703. Mean of distribution = 0.056  Alpha = 2.5; Beta = 96.5. Mean of distribution = 0.025 | Sousa (2013)  Edvardsson (2010)  Brignole (2006)  Farwell (2004) |
| Probability of carotid sinus massage | Beta | Alpha = 7; Beta = 238. Mean of distribution = 0.029  Alpha = 205; Beta = 365. Mean of distribution = 0.36  Alpha = 6; Beta = 1121. Mean of distribution = 0.005  Alpha = 112; Beta = 633. Mean of distribution = 0.15 | Sousa (2013)  Edvardsson (2010)  Baron-Esquivas (2010)  Brignole (2006) |
| Probability of carotid echo-Doppler | Beta | Alpha = 31; Beta = 214. Mean of distribution = 0.127  Alpha = 33; Beta = 712. Mean of distribution = 0.044  Alpha = 5; Beta = 93. Mean of distribution = 0.051 | Sousa (2013)  Brignole (2006)  Farwell (2004) |
| Probability of tilt test | Beta | Alpha = 39; Beta = 206. Mean of distribution = 0.159  Alpha = 201; Beta = 369. Mean of distribution = 0.353  Alpha = 96; Beta = 649. Mean of distribution = 0.129 | Sousa (2013)  Edvardsson (2010)  Brignole (2006) |
| Probability of ambulatory Holter monitor | Beta | Alpha = 29; Beta = 216. Mean of distribution = 0.118  Alpha = 67; Beta = 503. Mean of distribution = 0.118  Alpha = 28; Beta = 70. Mean of distribution = 0.286 | Sousa (2013)  Edvardsson (2010)  Farwell (2004) |
| Probability of hypertension map | Beta | Alpha = 17; Beta = 228. Mean of distribution = 0.069 | Sousa (2013) |
| Probability of basic lab tests | Beta | Alpha = 488; Beta = 82. Mean of distribution = 0.856  Alpha = 263; Beta = 482. Mean of distribution = 0.353 | Edvardsson (2010)  Brignole (2006) |
| Probability of ambulatory ECG monitoring | Beta | Alpha = 382; Beta = 188. Mean of distribution = 0.67  Alpha = 11; Beta = 87. Mean of distribution = 0.112 | Edvardsson (2010)  Farwell (2004) |
| Probability of electrophysiology testing | Beta | Alpha = 144; Beta = 426. Mean of distribution = 0.253  Alpha = 22; Beta = 723. Mean of distribution = 0.03  Alpha = 1.5; Beta = 97.5. Mean of distribution = 0.015 | Edvardsson (2010)  Brignole (2006)  Farwell (2004) |
| Probability of coronary angiography | Beta | Alpha = 133; Beta = 437. Mean of distribution = 0.233  Alpha = 12; Beta = 733. Mean of distribution = 0.016 | Edvardsson (2010)  Brignole (2006) |
| Probability of orthostatic blood pressure movements test | Beta | Alpha = 275; Beta = 295. Mean of distribution = 0.482  Alpha = 56; Beta = 1161. Mean of distribution = 0.046 | Edvardsson (2010)  Baron-Esquivas (2010) |
| Probability of neurological / psychiatric evaluation | Beta | Alpha = 270; Beta = 300. Mean of distribution = 0.474 | Edvardsson (2010) |
| Probability of basic blood chemistry | Beta | Alpha = 854; Beta = 363. Mean of distribution = 0.702 | Baron-Esquivas (2010) |
| Probability of enzymes | Beta | Alpha = 368; Beta = 849. Mean of distribution = 0.302 | Baron-Esquivas (2010) |
| Probability of brain CT scan | Beta | Alpha = 109; Beta = 1108. Mean of distribution = 0.0896  Alpha = 8; Beta = 90. Mean of distribution = 0.0816 | Baron-Esquivas (2010)  Farwell (2004) |
| Probability of thorax CT scan | Beta | Alpha = 13; Beta = 1204. Mean of distribution = 0.0107 | Baron-Esquivas (2010) |
| Probability of chest x-ray | Beta | Alpha = 632; Beta = 585. Mean of distribution = 0.519  Alpha = 87; Beta = 658. Mean of distribution = 0.117 | Baron-Esquivas (2010)  Brignole (2006) |
| Probability of abdominal echography | Beta | Alpha = 18; Beta = 727. Mean of distribution = 0.024 | Brignole (2006) |

* Distribution parameters estimated by assuming a 95% confidence interval based on mean value ± 30%.
